# Identification of Drug-Disease Associations Using Information of Molecular Structures and Clinical Symptoms via Deep Convolutional Neural Network

**DOI:** 10.3389/fchem.2019.00924

**Published:** 2020-01-10

**Authors:** Zhanchao Li, Qixing Huang, Xingyu Chen, Yang Wang, Jinlong Li, Yun Xie, Zong Dai, Xiaoyong Zou

**Affiliations:** ^1^School of Chemistry and Chemical Engineering, Guangdong Pharmaceutical University, Guangzhou, China; ^2^School of Chemistry, Sun Yat-Sen University, Guangzhou, China; ^3^Key Laboratory of Digital Quality Evaluation of Chinese Materia Medica of State Administration of Traditional Chinese Medicine, Guangzhou, China

**Keywords:** convolutional neural network, deep learning, drug-disease associations, fingerprint, symptoms

## Abstract

Identifying drug-disease associations is helpful for not only predicting new drug indications and recognizing lead compounds, but also preventing, diagnosing, treating diseases. Traditional experimental methods are time consuming, laborious and expensive. Therefore, it is urgent to develop computational method for predicting potential drug-disease associations on a large scale. Herein, a novel method was proposed to identify drug-disease associations based on the deep learning technique. Molecular structure and clinical symptom information were used to characterize drugs and diseases. Then, a novel two-dimensional matrix was constructed and mapped to a gray-scale image for representing drug-disease association. Finally, deep convolution neural network was introduced to build model for identifying potential drug-disease associations. The performance of current method was evaluated based on the training set and test set, and accuracies of 89.90 and 86.51% were obtained. Prediction ability for recognizing new drug indications, lead compounds and true drug-disease associations was also investigated and verified by performing various experiments. Additionally, 3,620,516 potential drug-disease associations were identified and some of them were further validated through docking modeling. It is anticipated that the proposed method may be a powerful large scale virtual screening tool for drug research and development. The source code of MATLAB is freely available on request from the authors.

## Introduction

Traditional drug development usually follows this paradigm of one drug, one gene, one disease, which is an expensive and time-consuming process with stunningly high failure rate. By conservative estimates, it takes about 15 years and $0.8–1.5 billion to bring a drug to market (Dudley et al., 2011; Yu et al., 2015), and during the development stage, almost 90% of the small molecules cannot pass the Phase I clinical trial and finally be eliminated (Wu et al., 2019). On the other hand, disease burden is increasing globally due to the growth of population, outbreak of infectious disease and emergence of antibiotic resistance (Shameer et al., 2018). In order to circumvent this dilemma, drug repositioning has become a promising alternative strategy for drug research and development (Wu et al., 2017).

Drug repositioning, also known as drug repurposing, drug reprofiling, and drug redirecting, which aims to find potential new indication for existing drug and apply the drug to the treatment of disease other than the drug's originally intended disease (Luo et al., 2016). It offers a possible way to greatly save time and cost, especially improve success rate, because of existing pharmacological and toxicological properties, as well as safety on known drug (Wang et al., 2014; Sun et al., 2017). However, successful drug repurposing stories are rare and rather random events (Sun et al., 2017). Well-known examples are sildenafil (trade name Viagra) and minoxidil, which were originally used to treat angina and hypertension. At present, they have been repurposed for the treatment of erectile dysfunction and hair loss, due to accidental discovery with a bit of luck (Bovac, 2013; Varothai and Bergfeld, 2014; Wu et al., 2019). Weakness of drug reprofiling is that it relies mainly on prior knowledge and clinical trials (Park et al., 2017), which is unfeasible in general and much too expensive to be applied on a large scale (Sun et al., 2017). For example, there are 2,593 approved small molecule drugs in DrugBank (Wishart et al., 2018) and 19,941 disease entries in MalaCards (Rappaport et al., 2017), resulting in more than 50 million of drug-disease combinations. Undoubtedly, it is almost impossible to effectively validate all possible associations of drug-disease through laboratory works and clinical trials. Therefore, it is urgent to develop *in silico* drug redirecting approaches for discovering new indications for approved drugs on a large scale.

Fortunately, with the accumulation of drug and disease related data, as well as the development of machine learning, numerous theoretical methods have been proposed to find new indications of drugs by identifying potential drug-disease associations. These computational approaches can be roughly divided into three mainstreams: drug-based, disease-based, and network-based. The former two are according to the assumption that drugs having similar structures/properties are inclined to be associated with diseases having similar pathogenesis/symptoms, and vice versa (Liu et al., 2016; Wu et al., 2017; Shameer et al., 2018). For example, Gottieb et al. (2011) utilized multiple drug-drug and disease-disease similarity measures for the prediction of drug repurposing using the logistic regression classifier. Zhu and Zhu (2015) introduced a method to identify repositioned drug for breast cancer by integrating the breast cancer survival data with the drug sensitivity information. By integrating information of drug chemical substructure, target domain and annotation, a novel method was presented to predict drug-disease associations based on the Laplacian regularized sparse subspace learning (Liang et al., 2017). Based on the drug features and disease semantic information, Zhang et al. (2018a) proposed a similarity constrained matrix factorization method for the prediction of drug-disease associations. Khalid and Sezerman (2018) combined the biological pathways, binding site structural similarities, disease-disease similarity with logistic regression classifier to predict approved and novel drug-disease associations. Wang et al. (2013) trained a support vector machine model to identify potential drug-disease interactions by integrating molecular structure, molecular activity and phenotype data. A support vector machine model was also built by Moghadam et al. (2016) to recognize novel drug indications through adopting kernel fusion technique and various features of drug and disease. By considering information of drug chemical structures, drug targets and gene expression patterns, Napolitano et al. (2013) also developed a support vector machine classifier to predict novel drug-disease associations.

The last one is based on the principle of “guilt-by-association” that drugs treating with same disease share structure/network properties and the diseases treated with the same drug also share phenotype/network properties (Wu et al., 2017). For instance, Zhao and Li (2012) defined a network-based gene closeness profile to relate drug to disease. Then, a Bayesian partition method was utilized to elucidate drug-disease associations by identifying drug-gene-disease co-modules. Huang et al. (2013) combined three different networks with edge weights of drug, genomic and disease phenotype, and developed network propagation approach to infer the drug-disease associations. Oh et al. (2014) constructed an integrative genetic network including protein-protein interaction network and gene regulatory network. Then, the distance between topology drug-module and disease-module were adopted as features for the prediction of novel drug-disease associations based on the random forest algorithm. A causal network connecting drug-target-pathway-gene-disease was built by Yang et al. (2014) who calculated the association scores between drugs and diseases by evaluating a drug's effects on multiple targets and pathway. Finally, probabilistic matrix factorization models were learned to identify therapeutic associations. Based on the propagation flow algorithm, Martinez et al. (2015) developed DrugNet for prioritization of drug-disease relationships through a network of interconnected drugs, proteins and diseases. A novel methodology was proposed by Yu et al. (2015) to discover the drug-disease associations by constructing heterogeneous network consisting of drugs, protein complexes and diseases. By building a heterogeneous network including drug-drug similarity network, disease-disease similarity network and known drug-disease association network, Liu et al. (2016) proposed a two-pass random walks with restart to predict new indications for approved drugs. Yu et al. (2016) represented a cluster method for prediction of new drug indications by using the identified drugs and disease modules based on the constructed drug network and disease network. Wu et al. (2017) constructed a novel weighted drug-disease pair network, where a node is a drug-disease pair and a weighted edge represents the node-node relation. Then, a semi-supervised graph cut algorithm was adopted to identify the potential drug-disease treatment interactions. Drug-disease associations were formulated as a bipartite network, Zhang et al. (2018b) presented the network topological similarity-based inference method to predict unobserved drug-disease associations based on the linear neighborhood similarity. Wu et al. (2019) introduced a method to detect drug-disease treatment relations by using drug-disease, drug-protein and disease-protein interaction data based on the random forest algorithm. By considering network similarities of drugs and diseases, Cui et al. (2019) proposed a novel method to predict drug-disease interactions based on the Gaussian interaction profile kernels and L_2,1_-norm.

Despite progresses in the past decade on identification of drug-disease associations, accurate prediction of treatment relations is still far from satisfaction. The first one recognized limitation, especially for the drug-based and disease-based methods, is the lack of a uniform or universal definition for calculating similarity, resulting in dramatical change of similarity score from one method to another. The second one, especially for network-based methods, is only fit for drugs or diseases included in the built network or dataset. Hence, some methods usually fail to discover the novel drugs and new indications. The third one is the restriction of only semantic similarity for assessing disease similarity based on the human phenotype ontology (Groza et al., 2015) and disease ontology (Bello et al., 2018). However, precise semantic relationships are not often captured (Zhu et al., 2013). Others or new attributes, such as symptoms (i.e., clinical manifestations information) may be adopted to characterize the disease, because symptoms are the most directly observable characteristics of a disease and the basis of clinical disease classification (Zhou et al., 2014), as well as diseases with similar symptoms usually share common genetic mechanisms (Xu et al., 2013). In addition, deep learning has been widely used in various research fields as a modern machine learning technique (Bai et al., 2019; Li et al., 2019; Steuer et al., 2019). However, its effectiveness for drug-disease associations prediction has not been evaluated.

In view of these reasons, a novel computational method was developed to identify drug-disease associations based on the information of molecular structures and clinical symptoms through deep learning method. Instead of using the information of drug related side effects, activity, target protein and their interactions, as well as disease-related human phenotype ontology, disease ontology, gene ontology and disease genes, the chemical fingerprints and disease symptoms were only utilized for enhancing the generalization ability. A novel two-dimensional matrix was constructed to characterize drug-disease association by considering the information of drug and disease, simultaneously. Finally, deep convolutional neural network was employed to construct model for identifying potential drug-disease associations.

## Materials and Methods

### Collection of Drug-Disease Associations

In order to construct a comprehensive and high-quality dataset of drug-disease associations, firstly, we downloaded the information of drug-disease associations contained in the file CTD_chemiclas_diseases.tsv from the Comparative Toxicogenomics Database (CTD, Ver. Feb, 2017) (Davis et al., 2019), which is a robust, publicly available database and provides manually curated information about chemical, gene, protein, disease and their relationships. Secondly, removed drug-disease pairs without annotation “therapeutic” in the field of Direct Evidence and with annotation “drug combination” in the field of Chemical Name, meaning that the obtained drugs itself have therapeutic effects on diseases, rather than exert functions by combining with other drugs. Thirdly, deleted drug-disease associations in which drugs had no information of CID numbers and SMILES (canonical simplified molecular input line entry system) strings in the PubChem database (Kim et al., 2016). Fourthly, canceled drug-disease pairs in which diseases were un-included in the work of Zhou et al. (2014). Finally, 26,521 drug-disease associations containing 4,501 drugs and 2,093 diseases were obtained (Supporting Information 1). These retrieved drug-disease associations were considered as positive examples.

The goal of current research is to identify potential therapeutic relationships from tremendous combinations between drugs and diseases based on deep learning method. This is a binary classification problem, therefore, it is necessary to build negative examples (i.e., drug-disease non-association pairs). Unfortunately, there is no database dedicated to collecting drugs without treatment relationships for diseases due to lack of research and application value. Consequently, we had to use the following strategy to produced negative samples: (1) Randomly selected drug and disease from positive samples to form new drug-disease association pair. (2) Eliminated the new association pair if it existed in the downloaded file CTD_chemiclas_diseases.tsv, otherwise, considered it as a negative sample. (3) Repeated steps (1) and (2), until the number of negative samples equals the number of positive samples.

Finally, a benchmark dataset with equal size of true drug-disease associations and false drug-disease associations was established. The “1:1” ratio can overcome the limitation of a larger number of negative examples and lead to unbiased prediction.

### Characterization of Drug-Disease Associations

**Figure 1:**
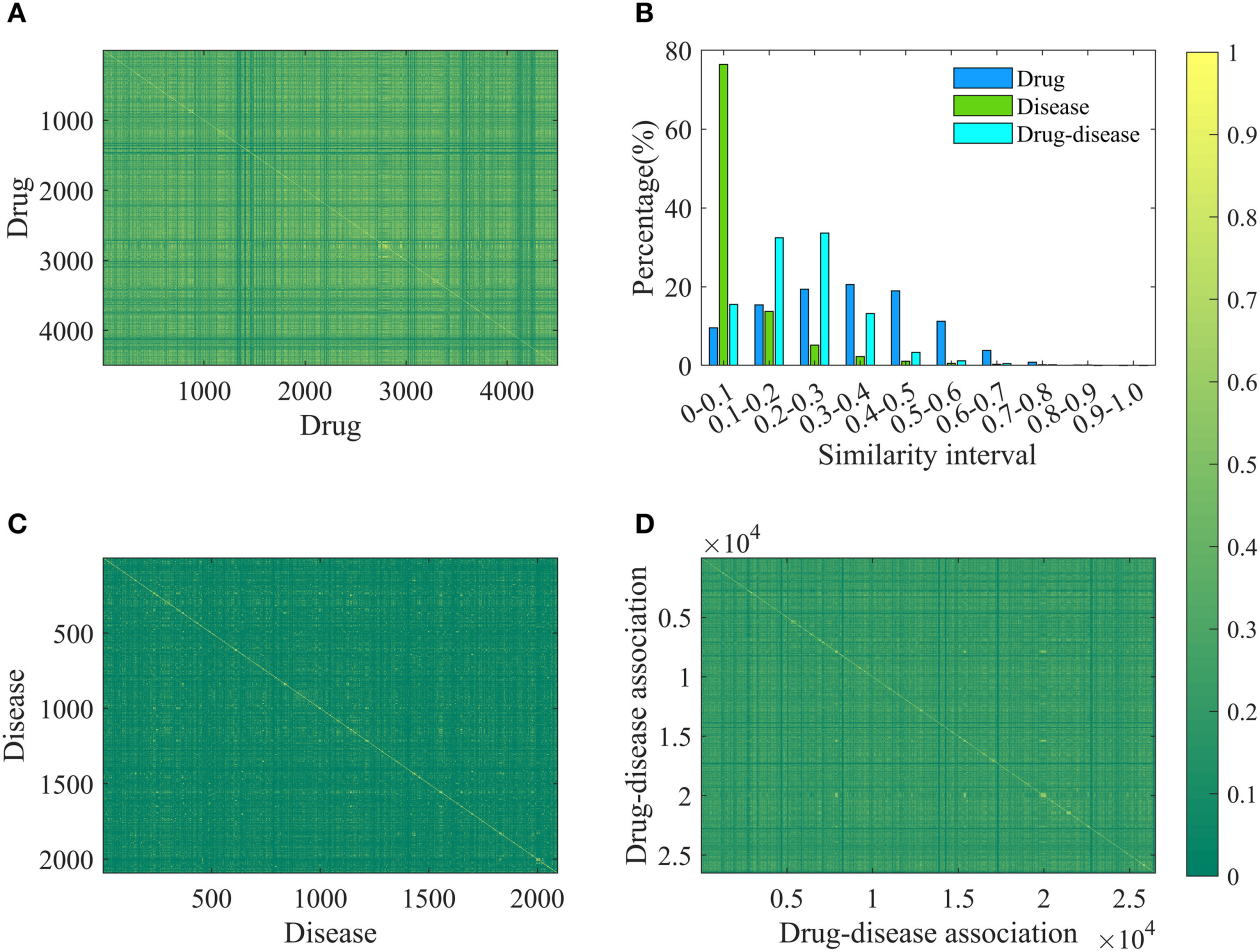
The results of drug, disease and drug-disease association similarity. **(A)** The calculation results of any two drug similarity. The 4501-by-4501 grid of pixels where 4501 is the number of drugs. Each pixel represents a similarity of two drugs and has a different color changing from green (0) to yellow (1). “1” represents that the structures of two drugs are exactly the same, and “0” means that their structures are completely different. **(B)** The statistical results of similarity of drug, disease and drug-disease association. **(C)** The calculation results of any two disease similarity. **(D)** The calculation results of any two drug-disease associations.

In order to increase the applicability of the current method, the Pubchem molecular fingerprint descriptor was calculated to characterize drug molecule by using the information of SMILES format and PaDEL-descriptor software (Yap, 2011). This fingerprint descriptor is a binary feature vector with 881 dimensions, in which every element corresponds to one specific chemical substructure and is encoded as either 1 or 0 to show clearly whether the substructure is contained in the drug molecular. For simplicity, the molecular fingerprint descriptor of drug *i* is represented by *F*_*i*_, _n_ (*n* = 1,2,…….,881). The molecular fingerprint is a simple but effective descriptor in the wide use of quantitative structure-activity relationship (Banerjee and Preissner, 2018; Zheng et al., 2019). Based on the fingerprint descriptor, the similarity of any two drug molecules was evaluated by calculating the Jaccard similarity coefficient (Levandowsky and Winter, 1971; Fuxman Bass et al., 2013; Li et al., 2016). Similarity values and statistical results are shown in Figures 1A,B. Clearly, the similarity values are in the range of 0–0.9956, about 10, 15, 19, 20, 18, and 11% are located in the range of [0–0.1], [0.1–0.2], [0.2–0.3], [0.3–0.4], [0.4–0.5], and [0.5–0.6], suggesting that drug molecular structures are very diverse and complex.

For each disease, the symptom information was retrieved from the human symptoms-disease network (Zhou et al., 2014), resulting in 105,892 connections between 2,093 diseases and 322 symptom terms. The associations between diseases and symptoms were acquired based on the co-occurrence of disease terms and symptom terms in the MeSH metadata field of PubMed and quantified using the term frequency-inverse document frequency. Therefore, a disease can be characterized by a feature vector with 322 dimensions, in which every element corresponding to one specific symptom and is encoded as a value larger than or equal to zero to explain the strength of the association between disease and symptom. For convenience of description, the symptomatic feature of disease *j* is characterized by *D*_*j*_, _m_ (m = 1,2,……,322). This representation is reasonable and based on this fact that many symptoms are not always present for a disease and occur with varying frequency. For any two diseases, we calculated the cosine value of the included angel between corresponding two symptom feature vectors to assess disease diversity. The statistical results and cosine values were shown in Figures 1B,C. It is clear that most (about 76%) of the cosine values are lower than 0.1, revealing that diseases in the benchmark dataset belong to various categories.

Different from previous studies, a novel gray-scale image method was proposed to characterize drug-disease relationships by considering both drug and disease properties. For drug *i* and disease *j*, a two-dimensional matrix *FD*_*i*_*j* with 881 × 322 was constructed. Value of element located in the *n*-th row and *m*-th column was calculated according to the following Equation (1):

(1) $$\begin{array}{r} FD_{i,j}\left(n,m\right)=F_{i}\left(n\right)+D_{j}\left(m\right) \end{array}$$

Then, the matrix was mapped to a gray-scale image to characterize the relationship between drug *i* and disease *j*. The rationality of the method is rooted in the paradigm of “structure determines function” and the fact that the clinical manifestation of disease is symptoms, which are widely used in disease diagnosis, treatment and classification research. Therefore, we utilize molecular fingerprint descriptors to characterize drug chemical structure and provide information on its functions, as well as adopt symptom features to represent disease and provide information on its pathological mechanisms, respectively. The introduced method is helpful to elucidate the relationship between drug and disease at the level of molecular structure and clinical phenotype.

For any two drug-disease associations Dr_1_-Di_1_ and Dr_2_-Di_2_, their similarity is defined by the following Equation (2):

(2) $$\begin{array}{r} Sim_{\text{D}{\text{r}}_{1}-\text{D}{\text{i}}_{\text{1}},\text{D}{\text{r}}_{2}-\text{D}{\text{i}}_{2}}=\frac{Jac\left(\text{D}{\text{r}}_{1},\text{D}{\text{r}}_{2}\right)+Cos\left(\text{D}{\text{i}}_{1},\text{D}{\text{i}}_{2}\right)}{2} \end{array}$$

Where, *Jac*(Dr_1_, Dr_2_) and *Cos*(Di_1_, Di_2_) mean the Jaccard similarity coefficient and cosine value, respectively. According to the definition, the similarity value of two drug-disease pairs is always located in the range of 0 and 1, and the higher value means the more similar. The statistical results and similarity values between any two drug-disease associations were shown in Figures 1B,D. Clearly, about 98% of the similarity values are in the range of 0–0.5, revealing that the benchmark dataset is complex and contains various drug-disease associations.

### Construction and Assessment of Model

The goal of this work is to identify whether an unknown drug-disease association has a therapeutic relationship or not, which is a two-class classification problem. Hence, deep convolution neural network was utilized to discriminate potential drug-disease associations owing to the success in image recognition and biomedicine (Esteva et al., 2017; Pelt and Sethian, 2018; Sullivan et al., 2018). The architecture and parameters of deep convolution neural network were optimized based on experience, and listed in Table 1. In addition, we used the optimizer of stochastic gradient descent with momentum 0.9. Initial learning rate was 0.01 and reduced the learning rate by a factor of 0.1 every 10 epochs. Maximum number of epochs for training was set to 50 and used a mini-batch with 128 observations at each iteration. The default values were used for all other parameters and the program was executed based on the MATLAB software.

**Table 1:** The architecture and parameters of deep convolution neural network.

**Layer**	**Size**
Image input	881 × 322
Convolutional	32 filters with 5 × 5, stride 2 × 2
ReLU	–
Max pooling	2 × 2, stride 2 × 2
Convolutional	64 filters with 5 × 5, stride 2 × 2
ReLU	–
Max pooling	2 × 2, stride 2 × 2
Convolutional	128 filters with 5 × 5, stride 2 × 2
ReLU	–
Max pooling	2 × 2, stride 2 × 2
Fully connected	500, dropout = 0.5
Fully connected	500, dropout = 0.5
Fully connected	500, dropout = 0.5
Fully connected	500, dropout = 0.5
Fully connected	2
Softmax	_
Classification	2

In order to evaluate the performance of current method, 20,000 positive and negative samples were randomly chosen from the benchmark dataset to construct a training set, and the remaining positive and negative samples were used to build a test set. In addition to accuracy (AC), sensitivity (SE), specificity (SP), precision (PR) and Matthew's correlation coefficient (MCC), we also utilize receiver operating characteristic curve (ROC), precision recall (RE) curve (PRC) and corresponding area (ROCA and PRCA) to estimate the predictive ability of the model.

**Figure 2:**
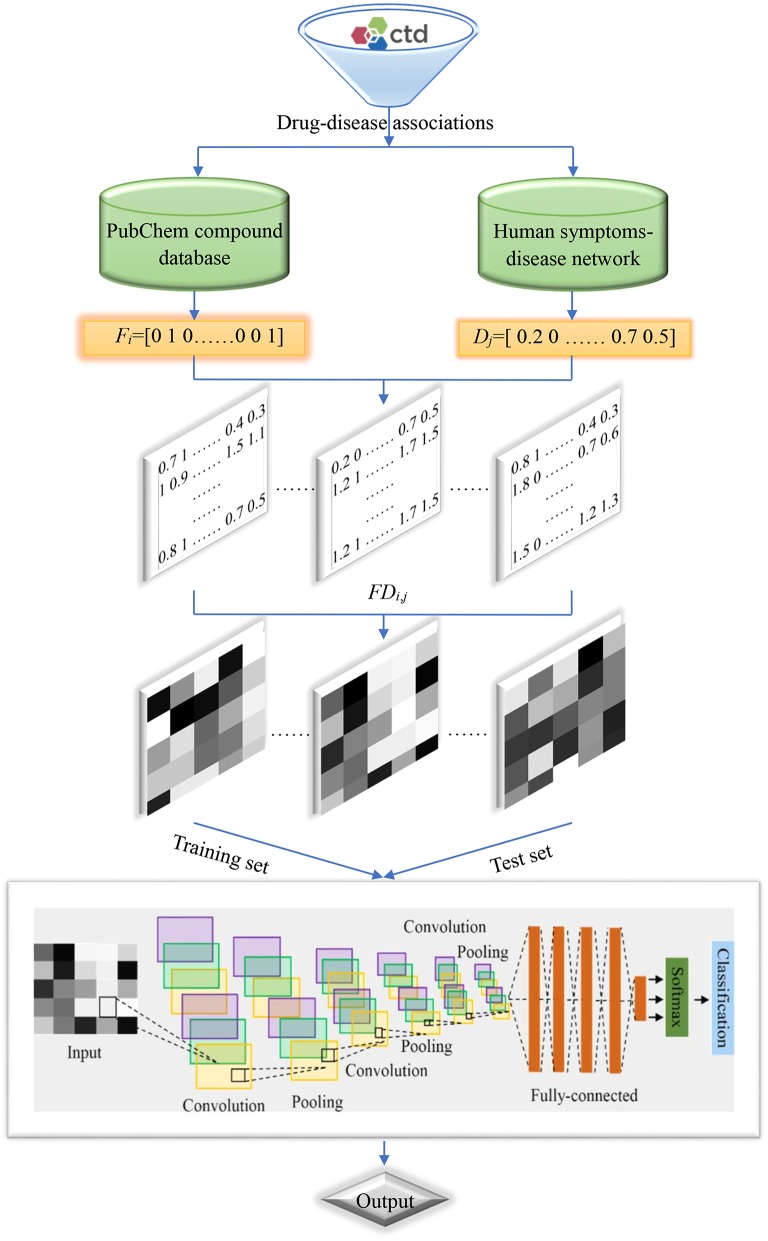
Flowchart of the current method.

Flowchart of the current method is shown in Figure 2, and detailed steps were described as follows:

Step 1. Retrieved drug-disease associations from the CTD database.
Step 2. Obtained the SMILES strings of drug molecules from the PubChem compound database and symptoms of diseases from the human symptoms-disease network, respectively.
Step 3. Produced molecular fingerprint descriptors and disease symptom features to characterize drugs and diseases, respectively.
Step 4. Generated two-dimensional matrixes and map it to gray-scale images to characterize drug-disease associations.
Step 5. Divided the benchmark dataset into training set and test set to build model and evaluate performance, respectively.

## Results and Discussion

### Performance Evaluation of Current Method

**Figure 3:**
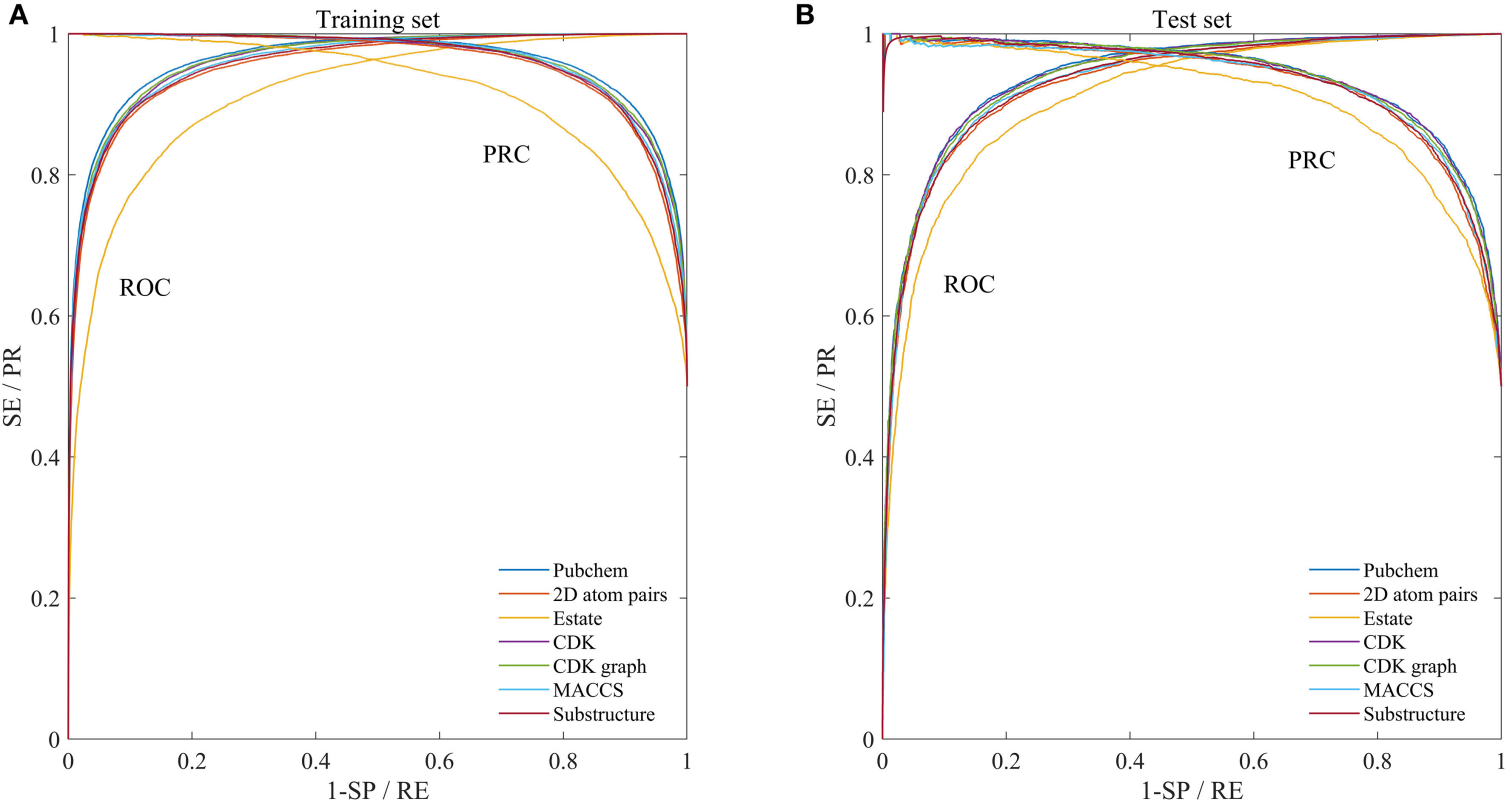
Mean ROC and PRC curves of execute 10 times based on the training set and test set. **(A)** Results of training set. **(B)** Results of test set.

In order to evaluate the performance for the negative sample random generation method, parallel experiments are performed 10 times for generating the negative samples, building the model and evaluating the performance. The statistical results of AC, SE, SP, PR, and MCC, as well as ROC and PRC derived from the training set and test set are shown in Figure 3 and listed in Table 2, respectively. For training set, average values of AC, SE, SP, PR, MCC, ROCA, and PRCA are 89.90, 88.96, 90.85, 90.67%, 0.7982, 0.9637 and 0.9651, with the relative standard deviations 0.30, 0.44, 0.16, 0.19, 0.66, 0.19, and 0.19%. For test set, average values and the corresponding relative standard deviations are 86.51 and 0.21%, 86.23 and 0.36%, 86.79 and 0.19%, 86.72 and 0.17%, 0.7302 and 0.50%, 0.9360 and 0.14%, 0.9352 and 0.17%, respectively. The AC, SE, SP, and PR from the training set and test set are higher than 85%. Meanwhile, the relative standard deviations are lower than 1%. These results reveal that the developed method can effectively capture information of drug-disease associations, and also has a strong robustness for generating negative samples and an outstanding ability to identify drug-disease associations.

**Table 2:** The statistical results of performed 10 times based on the training set and independent test set (Rsd: relative standard deviation).

**Dataset**	**Fingerprint**	**AC(%)/Rsd(%)**	**SE(%)/Rsd(%)**	**SP(%)/Rsd(%)**	**PR(%)/Rsd(%)**	**MCC/Rsd(%)**	**AUCR/Rsd(%)**	**AUCP/Rsd(%)**
Training set	Pubchem	89.90/0.29	88.96/0.44	90.85/0.16	90.67/0.19	0.7982/0.66	0.9637/0.19	0.9651/0.19
	2D atom pairs	88.73/0.18	87.36/0.23	90.10/0.17	89.82/0.17	0.7749/0.42	0.9531/0.08	0.9560/0.09
	Estate	83.51/0.27	80.22/0.40	86.79/0.18	85.86/0.21	0.6716/0.66	0.9106/0.25	0.9161/0.22
	CDK	88.66/0.78	87.97/1.0	89.37/0.58	89.22/0.63	0.7733/1.8	0.9557/0.59	0.9571/0.57
	CDK graph	89.28/0.78	88.55/0.33	90.02/0.23	89.87/0.24	0.7858/0.63	0.9606/0.18	0.9617/0.20
	MACCS	88.46/0.75	86.77/0.99	90.15/0.54	89.80/0.60	0.7696/1.7	0.9531/0.50	0.9520/0.43
	Substructure	88.83/0.10	87.38/0.14	90.28/0.07	89.99/0.08	0.7769/0.66	0.9558/0.06	0.9590/0.06
Test set	Pubchem	86.51/0.21	86.23/0.36	86.79/0.19	86.72/0.18	0.7302/0.50	0.9360/0.14	0.9352/0.16
	2D atom pairs	85.57/0.10	84.57/0.21	86.58/0.16	86.31/0.13	0.7116/0.24	0.9257/0.08	0.9261/0.09
	Estate	83.04/0.32	80.07/0.62	86.00/0.10	85.12/0.15	0.6619/0.79	0.9041/0.23	0.9057/0.21
	CDK	86.02/0.40	85.69/0.76	86.35/0.31	86.26/0.29	0.7204/0.96	0.9309/0.39	0.9304/0.22
	CDK graph	86.09/0.22	85.75/0.34	86.43/0.18	86.34/0.18	0.7218/0.53	0.9330/0.15	0.9326/0.22
	MACCS	85.17/0.38	83.92/0.79	86.43/0.35	86.08/0.29	0.7037/0.50	0.9217/0.25	0.9172/0.24
	Substructure	85.60/0.16	84.74/0.25	86.46/0.29	86.22/0.25	0.7121/0.38	0.9278/0.08	0.9283/0.10

### Comparison of Molecular Fingerprint Descriptors

In addition to the Pubchem fingerprint descriptor, we also calculated six kinds of fingerprint descriptors such as 2D atom pairs, Estate, CDK, CDK graph, MACCS, and Substructure (their detailed description can refer to the help file of PaDEL-descriptor). Then the model was constructed and evaluated based on the benchmark dataset. The statistical results, ROC and PRC were illustrated in Figure 3 and listed in Table 2, respectively.

We can see that the Estate descriptor achieve the lowest average AC, SE, SP, PR, MCC, AUCR, and AUCP for both the training set and test set, which may be caused by the fact that the descriptor has only a 79-dimensional feature vector and cannot adequately describe the molecular structure information. For 2D atom pairs, CDK, CDK graph, MACCS and Substructure, AC from the training set and the test set are about 89 and 86%, about 0.9 and 0.5% lower than those of Pubchem, respectively. For AC from the training set and test, statistical hypothesis tests including Wilcoxon rank sum test and two-sample Kolmogorov-Smirnov test between Pubchem and other descriptors were performed, and the corresponding results were listed in Table 3. For Wilcoxon rank sum test, most of the *p*-values are <1.8 × 10^−4^, only *p*-value between the Pubchem and the CDK graph derived from test set is 1.309 × 10^−3^. All *p*-values indicate that significant differences existed in the AC from Pubchem and other six descriptors. For two-sample Kolmogorov-Smirnov test, the lowest and highest are 3.286 × 10^−5^ and 9.050 × 10^−3^, respectively. All these *p*-values show significant differences. Therefore, Pubchem molecular fingerprint descriptor is the optimal feature for characterizing molecular structure in current research.

**Table 3:** The *p* values of hypothesis tests between Pubchem and other molecular fingerprint descriptors based on the AC.

	**Pubchem**	
	**Wilcoxon rank sum (Train/Test)**	**Kolmogorov-Smirnov (Train/Test)**
2D atom pairs	1.827e-04/1.817e-04	1.888e-05/1.888e-05
Estate	1.827e-04/1.817e-04	1.888e-05/1.888e-05
CDK	2.165e-05/7.361e-04	3.286e-05/9.050e-03
CDK graph	3.791e-04/1.309e-03	1.216e-03/6.899e-03
MACCS	1.827e-04/1.827e-04	1.888e-05/1.888e-05
Substructure	1.827e-04/1.827e-04	1.888e-05/1.888e-05

### Proportion of Positive and Negative Samples

**Figure 4:**
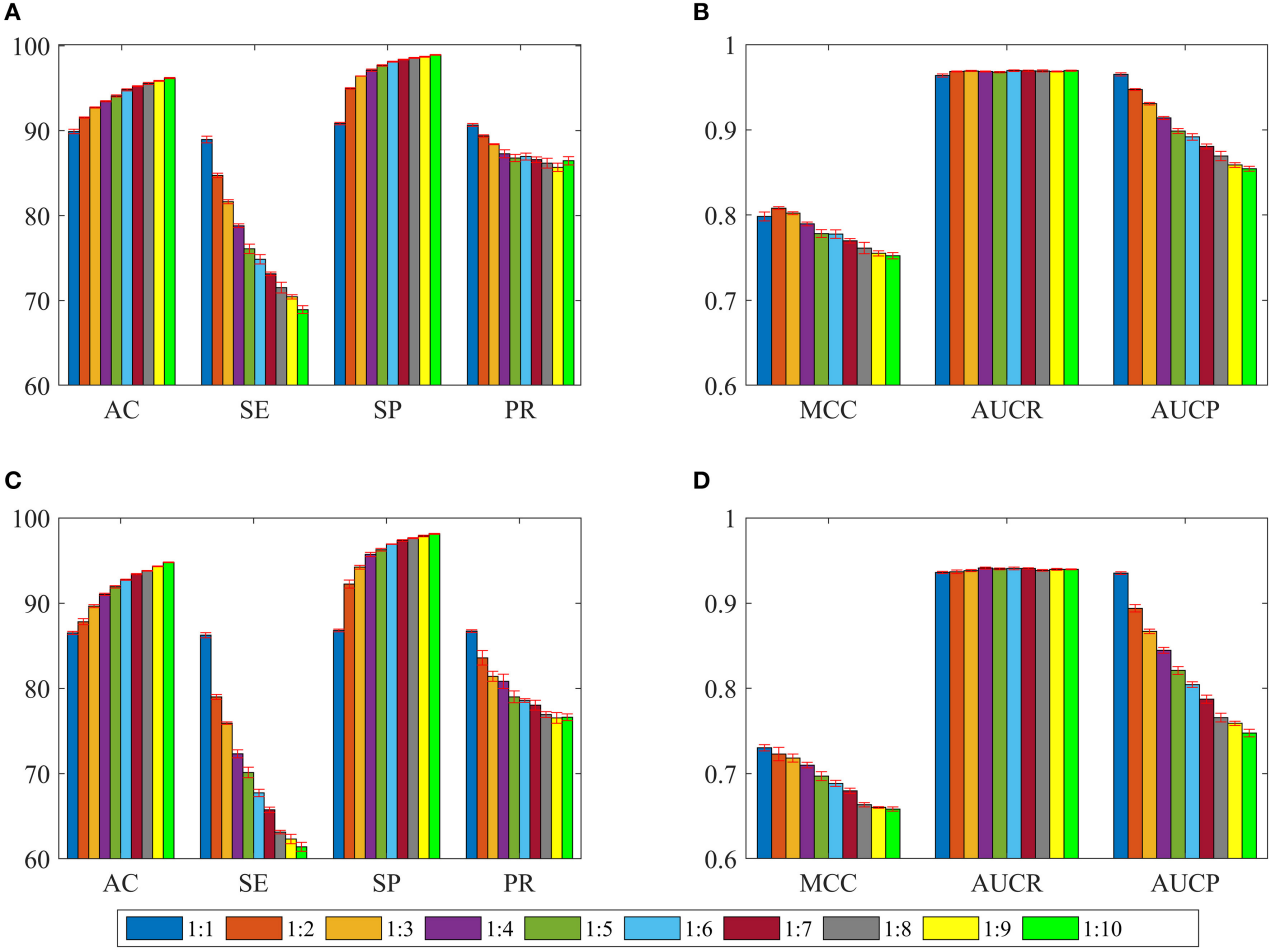
The statistical average results of various dataset, in which the ratios between positive and negative samples are 1:1, 1:2, 1:3, …, 1:10. The panels in **(A–D)** represent the mean values of AC, SE, SP, PR, MCC, AUCR, and AUCP, respectively. The red vertical bars indicate the standard deviations. **(A)** The statistical results of AC, SE, SP, and PR from training sets. **(B)** The statistical results of AUCR and AUCP from training sets. **(C)** The statistical results of AC, SE, SP, and PR derived from independent test sets. **(D)** The statistical results of AUCR and AUCP derived from independent test sets.

In order to overcome the problem of classification hyperplane skewness, a very common phenomenon in the field of machine learning, the ratio between positive and negative samples was set to 1:1. In fact, the number of negative samples is much larger than that of positive samples for identifying drug-disease associations. To assess the effect of positive and negative sample ratios on the performance of current method, we constructed a series of datasets in which the ratio was set to 1:2, 1:3, …, 1:10. Then, 3/4 of the positive and negative samples were randomly choose as the training set for building model, and the remaining positive and negative samples were considered as the test set for evaluating performance. The whole process was repeated five times, and the statistical mean results were display in Figure 4. For convenience of comparison, the statistical results in section of performance evaluation of current method also exhibited in Figure 4.

As shown in Figures 4A,C, average values of AC and SP increase slowly as the ratio changes from 1:1 to 1:10 for training sets and test sets. However, average values of SE and PR are slowly decreasing. For Figures 4B,D, we can see that average values of MCC and AUCP are also slowly decreasing as the ratios increase. The average values of AUCR fluctuate within a very small range. This result indicates that as the ratio improves, the number of negative samples in the training set dramatically increases and provide more negative sample information for training model, which makes the model easier to identify negative samples, but more difficult to identify positive samples. Although AC takes into account the prediction results of positive and negative samples simultaneously, its value is mainly determined by the prediction result of negative samples. Therefore, average values of AC improve as the ratios increase. On the contrary, average values of MCC and AUCP decrease. We also note that AUCR is insensitive to the ratio between positive and negative samples in the current research. Hence, it is reasonable to set the ratio of positive and negative samples to 1:1, which can ensure the model has high sensitivity, because the aim of our research is to identify potential drug-disease associations.

### Identification Power of New Indications for Existing Drugs

Finding new indications for marketable drugs can help pharmaceutical companies reduce costs and time. Our approach ability for drug repositioning was further estimated through generating new training set and test set based on the step-by-step strategy: (1) Randomly selected a positive sample (i.e., drug-disease association Dr_1_-Di_1_) to enter the training set. (2) Chose all positive samples including disease Di_1_ into the training set. (3) Repeated the steps 1 and 2, until the number of positive samples chosen reached 3/4 of all positive samples. The remaining 1/4 was entered into the test set. (4) Randomly selected a negative sample (i.e., drug-disease non-association pair NDr_1_-NDi_1_) into the training set. (5) All negative samples containing disease NDi_1_ were also entered into the training set. (6) Repeated steps 4 and 5 until the number of negative samples selected achieved 3/4 of all negative samples. The remaining 1/4 was contained in the test set.

**Figure 5:**
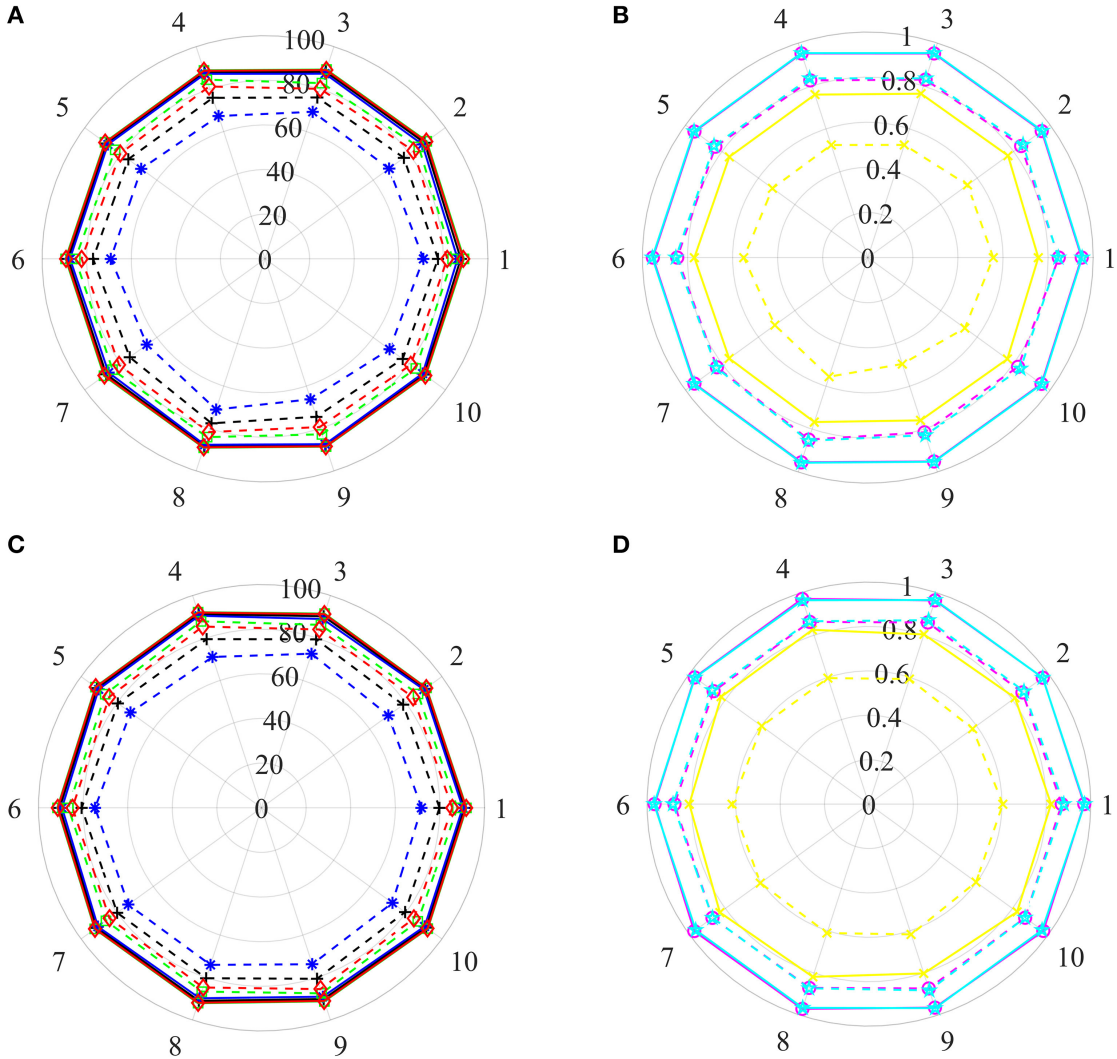
The prediction results of various training sets and independent test sets based on the step-by-step strategy. In polar coordinate system, radius is 100 **(A,C)** and 1 **(B,D)**, respectively. The solid and dashed lines represent the training set and the independent test set, respectively. Black+plus sign, blue+asterisk, green+ tetragonum, red+diamond, yellow+cross, magenta+circle and cyan+ pentagon mean the AC, SE, SP, PR, MCC, AUCR, and AUCP, respectively. **(A,C)** The results of AC, SE, SP, and PR. **(B,D)** The results of MCC, AUCR, and AUCP.

Based on the strategy, a disease is either involved in the training set or in the test set, which can guarantee disease information in the test set not existing in the training set. All of the above steps were repeated 10 times, 10 new training sets and corresponding test sets were then generated, and their prediction results were illustrated in Figures 5A,B.

For training sets, AC, SE, SP, and PR are located in the range of [87.82–88.47%], [86.39–87.64%], [88.52–89.65%], [88.39–89.39%], respectively. The MCC, AUCR, and AUCP change from 0.7567 to 0.7694, 0.9493 to 0.9547, 0.9512 to 0.9665, respectively. The corresponding relative standard deviations are 0.26, 0.42, 0.37, 0.33, 0.60, 0.18, and 0.20%, respectively. The average values are 88.14, 87.18, 89.10, 88.89%, 0.7630, 0.9519, and 0.9539, only about 1.76, 1.78, 1.75, 1.78%, 0.035, 0.012, and 0.011 lower than those of the training set derived from the benchmark dataset with the Pubchem descriptor (listed in Table 2).

For test sets, the minimum and maximum values of AC, SE, SP, PR, MCC, AUCR, and AUCP are 74.46 and 77.60%, 65.50 and 71.40%, 82.72 and 85.22%, 89.30 and 82.31%, 0.4960 and 0.5568, 0.8147 and 0.8486, 0.8279 and 0.8541, respectively. The corresponding relative standard deviations are 1.4, 2.7, 1.1, 1.2, 3.8, 1.4, and 1.1%, respectively. The average values are 76.28, 68.60, 83.97, 81.05%, 0.5320, 0.8340 and 0.8414, only about 10.23, 17.63, 2.82, 5.67%, 0.1982, 0.1290, and 0.0938 lower than those of the test set from the benchmark dataset with the Pubchem descriptor (listed in Table 2).

These results uncover that our method still obtains high predictive accuracy even though both training test and test sets are constructed rigorously, indicating that it has ability for identifying new drug indications.

### Recognition Ability of Potential Drug Molecules

Pharmaceutical companies are more interested in which drug or compound is effective on a new disease, i.e., whether this novel disease is associated with known or potential drug molecule. To this end, we appraise the performance of our method for identifying potential drug molecules or lead compounds by generating a serious of training test and test sets base on the step-by-step strategy mentioned above. In Steps 2 and 5, selected all positive examples including drug Dr_1_ and all negative examples comprising drug NDr_1_ into the training set and test set, respectively. The process was executed 10 times, 10 training sets and test sets were then obtained, and their prediction results were shown in Figures 5C,D.

For training sets, values of Acc, Sen, Spe, Pre, MCC, AUCR, and AUCP fluctuate from 90.08 to 91.50%, 88.91 to 90.63%, 91.16 to 92.37%, 90.96 to 92.23%, 0.8018 to 0.8301, 0.9638 to 0.9730, 0.9648 to 0.9725, respectively. The corresponding relative standard deviations and average values are 0.54 and 90.85%, 0.68 and 89.91%, 0.44 and 91.80%, 0.46 and 91.64%, 1.2% and 0.8172, 0.33% and 0.9691 and 0.25% and 0.9671, respectively. These results are very close to the results of the training set derived from the benchmark dataset with the Pubchem descriptor (listed in Table 2).

For test sets, values of Acc, Sen, Spe, Pre, MCC, AUCR,and AUCP are in the range of [78.51–80.61%], [70.34–74.61%], [86.14–87.84%], [83.96–85.51%], [0.5780, 0.6171], [0.8556, 0.8764] and [0.8642, 0.8829], respectively. the corresponding relative standard deviations and average values are 0.79 and 79.77%, 1.9 and 72.69%, 0.72 and 86.84%, 0.67 and 84.68%, 2.0 and 0.6015, 0.74% and 0.8675, 0.72% and 0.8732. Although the average Acc of test sets is about 10% lower than that of training sets, it is reasonable because the drug information in the test sets are excluded from the training sets. Therefore, these results suggest that current method can recognize candidate drugs or lead compounds with a high prediction accuracy.

### Prediction Capability of Potential Drug-Disease Associations

**Figure 6:**
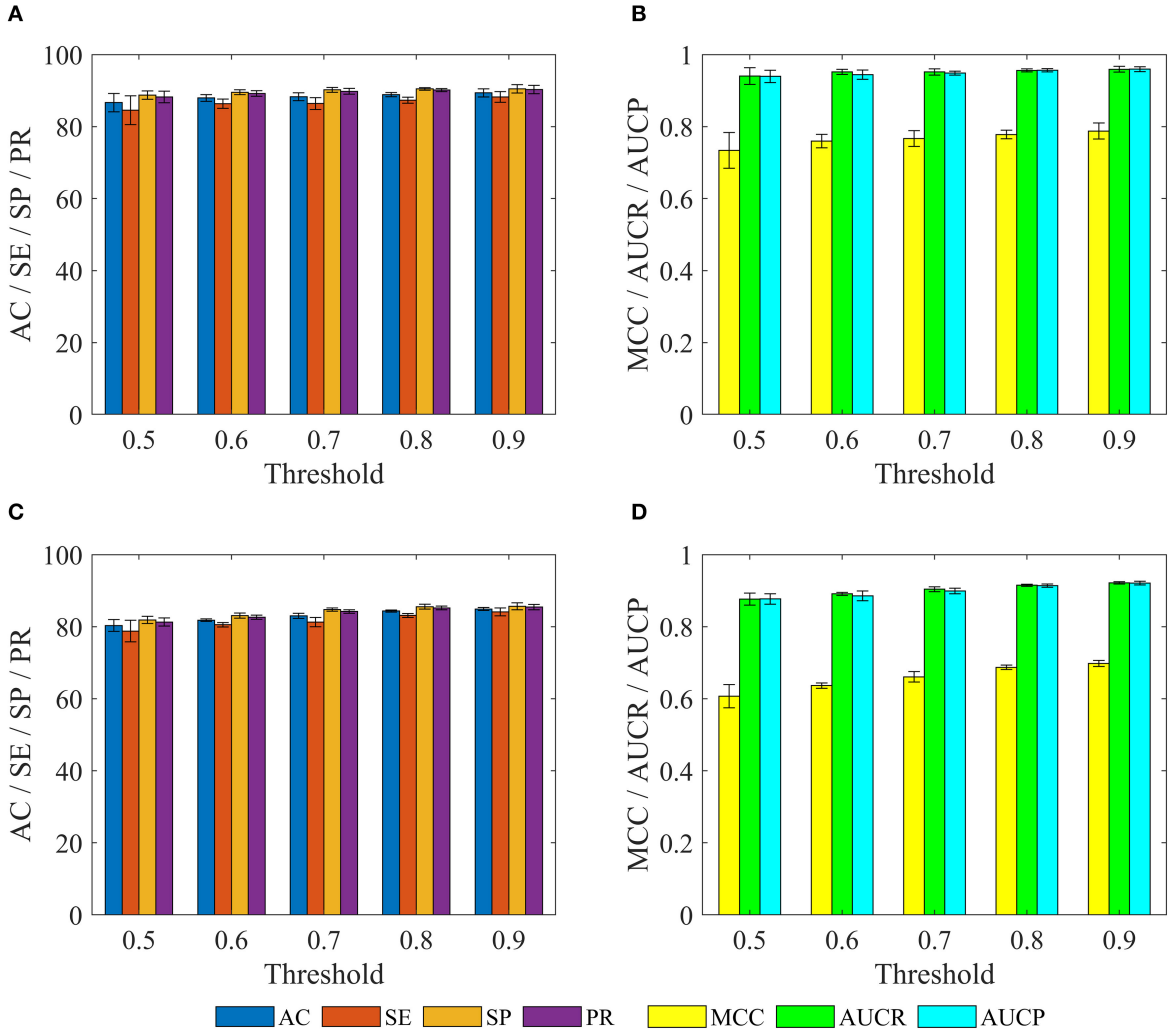
The statistical results of various training sets and independent test sets based on the different cutoffs. The panels in **(A–D)** indicate the mean values of AC, SE, SP, PR, MCC, AUCR, and AUCP, respectively. The black vertical bars indicate the standard deviations. **(A,B)** for training sets. **(C,D)** for independent test sets.

We further investigated performance for recognizing potential drug-disease associations by constructing a series of non-redundant benchmark dataset. Here, a non-redundant drug-disease associations database was constructed by randomly winnowing those association pairs that have more than a given threshold (i.e., similarity) to other pairs presented in the benchmark dataset. Then, 3/4 of positive and negative examples were adopted as training set to train model, and the remaining examples were utilized as test set to evaluate performance. The similarity threshold was set to 0.5, 0.6,…,0.9, and the construction of non-redundant dataset was repeated 10 times for each threshold, respectively. Note that thresholds of 0.1, 0.2, 0.3, and 0.4 were not employed, because the number of samples in the non-redundant dataset was too small to be statistically significant. The statistical results of training sets and test sets based on the various thresholds were shown in Figure 6.

For training set, we can observe that with the decrease of the threshold from 0.9 to 0.5, average values of AC, SE, SP, PR, MCC, AUCR, and AUCP are also gradually reduced from 89.37 to 86.65%, 88.22 to 84.56%, 90.51 to 88.74%, 90.29 to 88.21%, 0.7876 to 0.7339, 0.9592 to 0.9403, 0.9595 to 0.9396. The current method still obtains average accuracy of 86.65%, even if the threshold is reduced to 0.5.

For test set, average values of AC, SE, SP, and PR decrease gradually with the decrease of threshold from 0.9 to 0.5, but these values are consistently higher than 80% for threshold from 0.9 to 0.6. Meanwhile, average values of MCC, AUCR, and AUCP are always higher than 0.63, 0.89, and 0.88, respectively. Even when the threshold is decreased to 0.5, the current method still achieves the average AC of 80.33%, SE of 78.78%, SP of 81.88%, PR of 81.29%, Mcc of 0.6071, AUCR of 0.8766, AUCP of 0.8769.

These results demonstrate that the proposed method still achieves state-of-the-art performance, and has ability to identify novel drug-disease associations.

### Identify Ability of Independent Test Set

After evaluated the performance of the proposed method for identifying new indications, potential drugs and novel drug-disease associations, we further assessed the true predictive power based on the independent test set, which were generated by collecting drug-disease associations information from the CTD database (Ver. Jun, 2019). The independent test set contains 1,323 drug-disease associations (Supporting Information 2), which are not included in the benchmark dataset. In the new associations, drugs and diseases in 377 and 38 drug-disease pairs did not appear in the benchmark dataset, respectively.

The final identification model was built based on the all drug-disease associations in the benchmark dataset, and then used it to predictive the new drug-disease associations in the independent test set. We find that 973 drug-disease associations were correctly identified, and the prediction accuracy was 73.54% (973/1,323). For the 377 and 38 associations, 271 and 27 were correctly recognized, accuracy was 71.88% (271/377) and 71.05% (27/38), respectively.

These results reveal that our approach still achieves more than 70% prediction accuracy for these new drug-disease associations, indicating the reliability of the method.

### Large-Scale Prediction of Drug-Disease Associations

We further conducted a comprehensive and large-scale prediction for unknown drug-disease associations by using the final model. In order to generate the unknown associations, we firstly downloaded the information on structure and physicochemical properties of compounds/drugs from the DrugBank dataset. Secondly, deleted those compounds/drugs according to the Lipinski's rule of five (i.e., molecular mass <500 daltons, <5 hydrogen bond donors and 10 hydrogen bond acceptors, octanol-water partition coefficient logP <5). Thirdly, randomly combined the compounds/drugs collected from the DrugBank and diseases involved in the benchmark dataset. Finally, 24,266,646 unknown associations were generated. The final model identified 3,620,516 potential associations. We rank all the potential associations according to the probability in descending order, and the results show that 294,354 associations (Supporting Information 3) are the most likely to be putative associations because their probability values are higher than 0.99.

**Figure 7:**
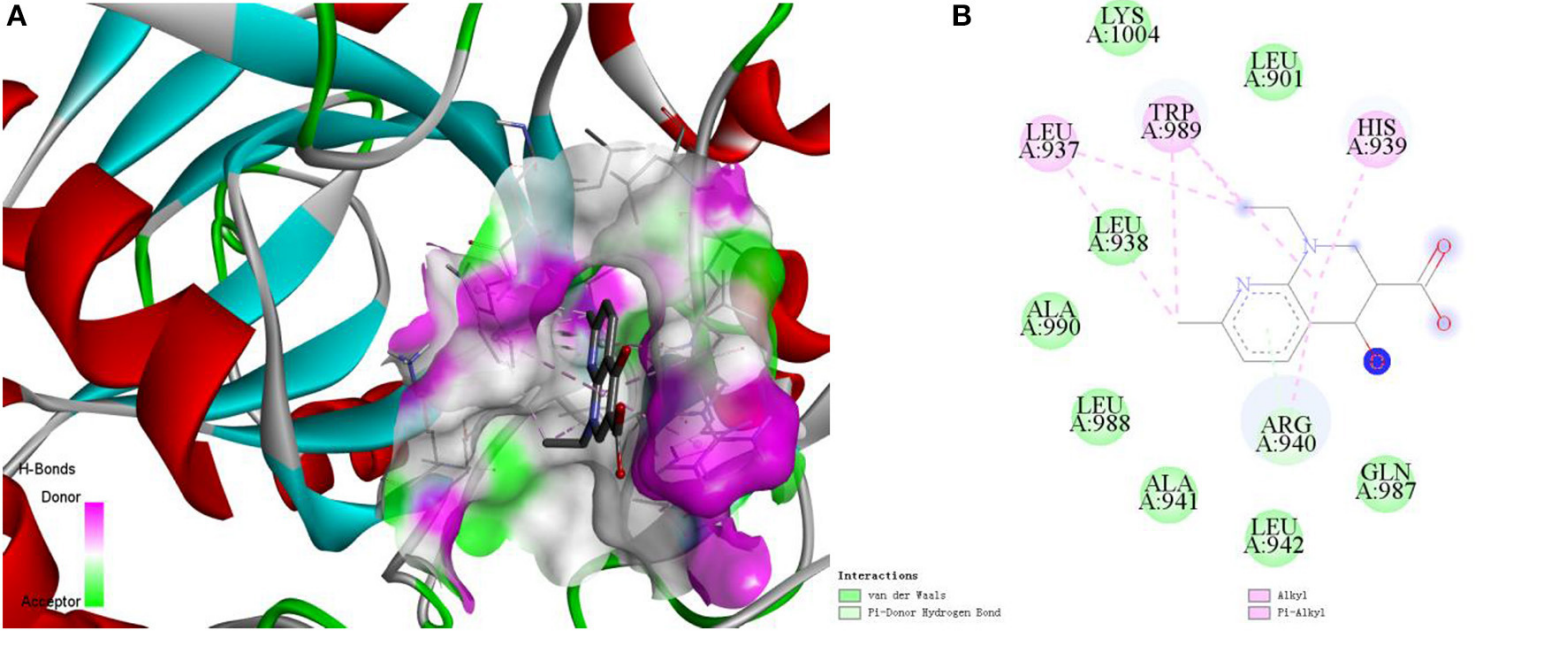
The complex model **(A)** of ligand compound (Pubchem CID: 4421) and receptor protein (UniprotKB: HAIR_HUMAN) as well as their interactions **(B)**.

Here, we take the one recognized associations as examples to illustrate the practical application of current method. Alopecia, also known as hair loss or baldness, refers to partial or complete loss of hair from part of the head or body. It usually can be classified into four types: male-pattern hair loss, female-pattern hair loss, alopecia areata and telogen effluvium, and the corresponding cause is genetics and male hormones, unclear, autoimmune, physically or psychologically stressful event (Vary, 2015). Although medications minoxidil, finasteride, and dutasteride have been used to treat hair loss, they have limited effects and can only prevent further baldness without regenerating lost hair (Rogers and Avram, 2008; Banka et al., 2013). The current method identified a possible association between the disease and compound nalidixic acid (Pubchem CID: 4421). The compound is a synthetic quinolone and composed of 1,8-naphthyridin-4-one substituted by carboxylic acid, ethyl and methyl groups at positions 3, 1, and 7, respectively. Some studies have shown that lysine-specific demethylase hairless (UniprotKB: HAIR_HUMAN) is a protein related with hair loss (Klein et al., 2002; Liu et al., 2014). Docking simulations between the compound and the protein was executed by using the AutoDock (Santos-Martins et al., 2014) program and DS visualizer software. The 3-dimensional structural information of protein was downloaded from the SWISS-MODEL Repository. The Lamarckian genetic algorithm was adopted to search the docking conformation. Finally, the optimized docking model with binding energy −6.26 kcal/mol and inhibition constant (Ki) 25.66 μM was obtained. Complex model of protein and compound as well as their interactions were displayed in Figures 7A,B.

We can observe that Var der Waals interactions exist between the compound and amino acid residues Leu901, Leu938, Ala941, Leu942, Gln987, Leu988, Ala990, and Lys1004. The small molecule is connected to the protein through π-donor hydrogen bond between six-membered ring and residue Arg940. Moreover, there are some hydrophobic interactions such as π-alkyl interactions between six-membered ring and residues His939, Arg940, and Trp989, alkyl interactions between the compound and residues Leu937 and Trp989. Therefore, we can assume that the compound may be used as a drug to treat hair loss through interacting with the target protein, which is worthy of further experimental verification.

## Conclusions

In this study, clinical manifestations information and molecule fingerprint descriptor are utilized to characterize disease and drug, respectively. A novel two-dimensional matrix is constructed and then map it into a gray-scale image to characterize drug-disease association. Deep convolutional neural network is introduced to construct model to identify potential drug-disease associations. The performance of current method is evaluated by building the benchmark dataset, and the optimal molecule fingerprint descriptor is determined by comparing with other various descriptors. In addition, the prediction ability of our method for identifying new drug indications, lead compounds, potential and true drug-disease associations has also been validated through a series of experiments. Finally, the practical application capability has been demonstrated by molecular simulation experiments. Our work gives a new insight for study of drug-disease associations at the level of disease clinical symptom and drug molecule structure. It is anticipated that the proposed method may be a powerful tool for new drug research and development.

## Data Availability Statement

Publicly available datasets were analyzed in this study. This data can be found here: http://ctdbase.org/.

## Author Contributions

ZL collected the data, experimented, and drafted the manuscript. QH provides experimental ideas and revised manuscripts. XC, YW, JL, YX, and ZD participated in the discussion of this work. ZL and XZ also participated in the discussion and revision of the manuscript. All authors read, commented, and approved the final manuscript.

### Conflict of Interest

The authors declare that the research was conducted in the absence of any commercial or financial relationships that could be construed as a potential conflict of interest.

## Supplementary Material

Supporting Information 1 The drug-disease associations retrieved from the CTD database.

Supporting Information 2 The independent test set.

Supporting Information 3 The most likely drug-disease associations.
